# Practical Considerations for the Diagnosis and Management of Isovaleryl-CoA-Dehydrogenase Deficiency (Isovaleric Acidemia): Systematic Search and Review and Expert Opinions

**DOI:** 10.3390/ijns11040092

**Published:** 2025-10-10

**Authors:** Eva Thimm, Anselma Riederer, Jerry Vockley, Dries Dobbelaere, Monique Williams, Anita MacDonald, Katharina Dokoupil, Ulrich A. Schatz, Regina Ensenauer

**Affiliations:** 1 Department of General Pediatrics, Neonatology, and Pediatric Cardiology, University Children’s Hospital, Faculty of Medicine, Heinrich Heine University Düsseldorf, 40225 Düsseldorf, Germany; eva.thimm@med.uni-duesseldorf.de; 2 Institute for Medical Information Processing, Biometry, and Epidemiology, Medical Faculty, Ludwig-Maximilians-Universität München, 81377 Munich, Germany; 3 Department of Pediatrics, University of Pittsburgh School of Medicine, Pittsburgh, PA 15224-1334, USA; vockleyg@upmc.edu; 4 Medical Reference Center for Inherited Metabolic Diseases, Jeanne de Flandre University Hospital and RADEME, Research Team for Rare Metabolic and Developmental Diseases, EA 59037 Lille, France; dries.dobbelaere@chu-lille.fr; 5 Center for Lysosomal and Metabolic Diseases, Department of Pediatrics, Erasmus Medical Center, 3015 CN Rotterdam, The Netherlands; williamsmonique59@outlook.com; 6 Dietetic Department, Birmingham Children’s Hospital, Birmingham B4 6NH, UK; anita.macdonald@nhs.net; 7 Department of Pediatrics, Dr. von Hauner Children’s Hospital, University Hospital, Ludwig-Maximilians-Universität München, 80337 Munich, Germany; katharina.dokoupil@med.uni-muenchen.de; 8 Gynecological Tumour Genetics, TUM University Hospital, Technical University of Munich, 81675 Munich, Germany; ulrich.schatz@mri.tum.de

**Keywords:** inborn error of metabolism, IVA, isovaleric acidemia, isovaleryl-CoA dehydrogenase, systematic search and review, expert opinion, newborn screening, disease spectrum

## Abstract

Isovaleric acidemia (IVA, OMIM 243500) is an inherited disorder of leucine metabolism caused by a deficiency of isovaleryl-CoA dehydrogenase (IVD), leading to an accumulation of isovaleric acid and its derivates 3-hydroxyisovaleric acid, isovaleryl (C5)-carnitine and isovalerylglycine in body fluids. The clinical presentation is highly variable, ranging from life-threatening metabolic crises with metabolic acidosis and hyperammonemia to a clinically asymptomatic only biochemical phenotype. Newborn screening for IVA has been established in many countries. Treatment consists of a protein-restricted diet combined with supplementation of carnitine and/or glycine and emergency treatment in catabolic episodes. Still, evidence-based recommendations for the diagnosis and management of IVA patients with various phenotypes are lacking. Therefore, a systematic search and review of the literature was conducted to make suggestions for the care of patients with IVA based on both the available scientific evidence and consensus-derived expert conclusions. Based on a comprehensive set of literature data published between 1966 and 2024, 15 statements were phrased on the presentation, diagnosis, management, and outcome of IVA involving clinical, biochemical, and nutrition expertise. These statements can serve as a basis for more standardized care for IVA.

## 1. Introduction

Isovaleric acidemia (IVA, synonym: isovaleric aciduria or isovaleryl-CoA dehydrogenase deficiency) is an autosomal recessive inborn error of leucine metabolism first described in 1966 in two siblings with mild mental retardation, recurrent episodes of ketoacidosis, and a characteristic body odor resembling “sweaty feet” [1].

The reported incidence of IVA worldwide is approximately 1 in 100,000 in screened patients and 1 in 280,000 in clinically diagnosed patients [2,3]. The higher incidence of IVA in screened populations indicates a significant proportion of asymptomatic patients [4,5].

The isovaleryl-CoA dehydrogenase (*IVD*) gene (EC 1.3.99.10) is located on chromosome 15q14-q15 and comprises 12 exons spanning approximately 15 kb of DNA [6]. IVD is a homotetrameric mitochondrial flavoprotein with a subunit size of 43 kDa [7]. It catalyzes the conversion of isovaleryl-CoA to 3-methylcrotonyl-CoA [1,8]. IVD deficiency causes the accumulation of isovaleric acid and its derivatives, including 3-hydroxyisovaleric acid (3-HIVA), isovaleryl (C5)-carnitine, and isovalerylglycine (IVG), in plasma, urine, and other body fluids.

The pathophysiology of IVA is not fully understood. Postulated mechanisms include impairment of the tricarboxylic acid cycle through the inhibition of citrate synthase and isocitrate dehydrogenase [9], increased oxidative stress [10], and hyperammonemia due to the inhibition of N-acetylglutamate synthase (NAGS) and acetyl-CoA depletion [11].

Patients with IVA exhibit a wide range of clinical presentations, ranging from severely affected to asymptomatic [12]. Historically, two phenotypes of IVA have been described: Patients with the “acute neonatal form” present in the first days of life with an episode of fulminant metabolic acidosis, ketosis, and hyperammonemia. Without prompt intervention, this condition can rapidly progress to coma and ultimately death [13,14]. In contrast, a “chronic intermittent form” has been defined as a clinical picture of failure to thrive and/or developmental delay, with or without recurring episodes of acidosis during periods of catabolic stress [1]. In clinical practice, the disease is best thought of as a continuum of severity. Diagnosis based on clinical symptoms is usually made in the first few years of life [15], but more moderate forms of IVA have been diagnosed in adults who may have gone undetected during infancy or early childhood [16]. Clinical course and outcome are not well predicted by the initial clinical presentation [17].

A clinically very mild or asymptomatic form of IVA was identified in a high percentage of patients diagnosed through newborn screening (NBS) [12,18]. Individuals with this type of IVA appear to have only a biochemical phenotype with no or very mild clinical symptoms. In these patients, concentrations of relevant metabolites in NBS are lower than in symptomatic patients [12,19,20,21]. Patients with IVA have therefore been classified as “metabolically severe” versus “metabolically mild or intermediate” [22] or, in a recent analysis of 84 screened patients, as “classical” versus “attenuated” [19].

More than 100 pathogenic *IVD* gene variants have been described in IVA patients (Human Gene Variant Database). No clear phenotype–genotype correlation has been observed [23,24,25,26], except for a “mild variant” (MANE [Matched Annotation from NCBI and EMBL-EBI] transcript: NM_002225.5:c.932C>T (p.Ala311Val)), also known as c.941C>T, p.Ala314Val, originally described as c.932C>T, p.Ala282Val [27], found in a homozygous or compound heterozygous state in a high percentage of asymptomatic individuals detected by NBS [12].

Treatment of IVA patients consists of a protein-restricted diet to reduce the production of isovaleryl-CoA from leucine catabolism, with supplementation of L-carnitine and/or L-glycine to improve the clearance of accumulating metabolites. In case of an impending catabolic crisis, emergency regimens are implemented with a high-calorie diet and reduced protein intake.

Data on the management of IVA patients are limited and mainly based on case reports. The rarity of the disease prevents individual treatment centres from gaining extensive first-hand experience of the full spectrum of the disease. Therefore, there is an urgent need for universal management guidelines and guidance, especially as IVA screening is included in NBS programmes in many countries worldwide.

Here, we present the results of (i) a systematic search and review of the literature on IVA and (ii) expert considerations for the management of patients covering the entire disease spectrum derived from the published evidence.

## 2. Methods

The expert author group included paediatric metabolic physicians, a medical geneticist, and specialist metabolic nutritionists. The group identified and agreed on a set of 22 structured key questions related to the clinical features, diagnosis, treatment, and monitoring, and outcome of IVA (Table S1).

A systematic search and review of the literature on IVA was conducted using Medline, Embase, the Cochrane Collaboration and Google Scholar for the years 1966 to 2024. The indexing terms used for the search are listed in Table S2. Included literature was reviewed by at least two members of the expert author group, and disagreements were resolved by discussion, before the conclusions were considered as evidence.

Due to the rarity of the condition, large randomized controlled trials are lacking for almost all aspects of IVA. Therefore, the level of evidence in the literature was often low. Because “recommendations” are considered evidence-based advice and good evidence was often lacking, the author group formulated “statements” that are based on both the available scientific evidence and consensus-derived expert conclusions from the literature.

## 3. Results of the Systematic Search and Review and Statements

### 3.1. Clinical Course

**Statement** **1.** *Isovaleric acidemia must be considered in the differential diagnosis in any neonate in poor clinical condition or with suspected sepsis, as well as in patients of any age presenting with faltering growth, developmental delay, and recurrent episodes of illness associated with ketoacidosis.*

The symptoms of IVA overlap with other intoxication-type inborn errors of metabolism and can occur at any age. IVA manifests either as episodic metabolic decompensation or as a more insidious chronic course. Classical triggers for metabolic decompensation involve any situation that induces catabolic stress or excessive protein intake [15,28]. As with other organic acidurias, a distinction has been made in IVA between “early-onset acute” and “late-onset chronic” presentation. The term “early diagnosis” includes patients diagnosed within the first 4 to 5 weeks of life, as published previously [15,28]. The term “late diagnosis” therefore refers to all patients diagnosed after this period.

#### 3.1.1. Early-Onset Acute Form

The neonate has a restricted range of responses to severe illness. Affected newborns typically exhibit symptoms such as poor feeding, vomiting, lethargy, and continuous clinical deterioration to coma or seizures during the first week of life. In addition, affected individuals may exhibit a characteristic “sweaty feet” body odor [15,28], which can serve as a clear distinguishing feature from other organic acidurias that otherwise have the same clinical symptoms. The initial metabolic decompensation in IVA is typically less severe than in the other classic organic acidemias. However, life-threatening metabolic crisis may occur [15,29]. Patients with a neonatal crisis due to inborn errors of metabolism are often difficult to distinguish from neonates with sepsis.

#### 3.1.2. Late-Onset Chronic Form

In late-onset patients, frequent symptoms are failure to thrive, developmental delay, or neurocognitive dysfunction, and intermittent episodes of illness, often triggered by infections with vomiting, ketoacidosis, and altered mental status [15,28].

#### 3.1.3. Symptoms in Patients Diagnosed by Newborn Screening

**Statement** **2.** *As a preventive measure, NBS reduces neonatal mortality in patients with classic IVA. At present, it is doubtful that identification of metabolically mild forms by NBS is of any clinical relevance.*

Most patients with IVA detected by NBS are asymptomatic at the time of diagnosis [12,18,22,30,31]. Among individuals with an NBS diagnosis, a metabolically mild form of IVA was identified in major proportions [12,18,19,22,32].

In early-onset patients, diagnosis and subsequent initiation of treatment have been shown to occur significantly earlier following diagnosis by NBS than after clinical diagnosis [18,28]. However, severely affected newborns may present with clinical symptoms before NBS results are available [5,18,29,32], as seen in patients with methylmalonic acidemia (MMA) or propionic acidemia (PA) [18,33,34]. An analysis of 24 patients with classic IVA showed a trend towards lower neonatal mortality of 3.8% when diagnosed by NBS [18] compared with a cohort of 155 clinically diagnosed patients (mortality 18.7%) [28].

#### 3.1.4. Long-Term Clinical Course

Clinical course and outcome cannot be well predicted by the initial presentation in symptomatic patients [17,35]. Following diagnosis, a broad clinical spectrum can ensue, ranging from recurrent episodes of metabolic derangement and severe mental handicap to an asymptomatic state. The frequency of symptomatic episodes was found to be highest during early infancy and to decrease with age [18,28,36]. Metabolic crises have rarely been described in adulthood [16,37]. Large longitudinal studies investigating the clinical course of patients under standardized treatment regimens are missing.

### 3.2. Diagnosis

#### 3.2.1. Baseline Laboratory Tests

**Statement** **3.** *Blood gases, lactate, ammonia, and ketone bodies should be measured if an organic acidemia including IVA is suspected.*

IVA can present with any of the biochemical findings shown in Table 1, commonly associated with organic acidurias [17,38]. Less common initial symptoms include severe hyperglycemia and ketoacidosis mimicking diabetic coma [39] or pancreatitis [40].

#### 3.2.2. Specialized Biochemical Investigations

**Statement** **4.** *Organic acids in urine and the acylcarnitine profile in blood should be measured if an organic acidemia including IVA is suspected.*

The hallmarks of IVA in blood and urine, respectively, are elevated isovaleryl (C5)-carnitine and IVG. In IVA, the concentrations of these metabolites are elevated regardless of the patient’s actual metabolic condition. Several additional organic acids can also be elevated and detected in urine, especially 3-HIVA. Isovaleric acid itself is not routinely measured due to its highly volatile nature. This organic acid, which is responsible for the distinctive body odor associated with the disorder, cannot be detected using conventional gas chromatography-based techniques.

#### 3.2.3. Differential Diagnosis

Differential diagnoses for IVA include multiple acyl-CoA dehydrogenase deficiency (MADD, glutaric aciduria type 2) and riboflavin deficiency or defects in riboflavin metabolism [41], as these conditions also exhibit elevated concentrations of isovaleryl-CoA derivatives. Nevertheless, diagnosis of IVA is typically straightforward, as MADD and disorders of riboflavin metabolism present with increased concentrations of multiple acylglycines and acylcarnitines that are not increased in IVA. Deficiency of 3-methylcrotonyl-CoA carboxylase is another disorder of leucine catabolism characterized by the excretion of high amounts of 3-HIVA [42]. However, in this disorder, the concentration of 3-methylcrotonylglycine is markedly elevated.

Isovalerylcarnitine cannot be distinguished from other 5-carbon unit acyl isomers, such as 2-methylbutyrylcarnitine and pivaloylcarnitine by direct flow injection mass spectrometry, without chromatographic separation of acylcarnitine isomers [43]. An increase in 2-methylbutyrylcarnitine concentrations is seen in individuals with 2-methylbutyrylglycinuria, a condition caused by short/branched-chain acyl-CoA dehydrogenase deficiency, which is an inherited disorder affecting isoleucine metabolism [44,45]. These patients excrete elevated concentrations of 2-methylbutyrylglycine in urine. Consequently, organic acid analysis provides essential Supplementary Information that is complementary to that obtained from acylcarnitine analysis.

Pivaloylcarnitine is derived from pivalic acid, which is included in several antibiotics to increase their intestinal absorption rate [43,46]. Pivalate derivatives are also utilized in the cosmetic industry as moisturizers [46,47]. Organic acids are normal in patients with elevated concentrations of pivaloylcarnitine [43].

In comparison to other classical organic acidemias such as PA and MMA, the findings of analysis of plasma amino acid profiles are less informative for the diagnosis of IVA. This is because glycine concentrations in IVA patients vary considerably and are frequently normal or only moderately increased at the time of diagnosis [48,49]. Presumably, glycine tends to decrease in IVA patients as a consequence of conjugation with isovaleryl-CoA [48]. Markedly elevated plasma glycine concentrations in patients with IVA often result from L-glycine supplementation during follow-up.

#### 3.2.4. Newborn Screening

**Statement** **5.** *Following a positive NBS result, the analysis of organic acids in urine (IVG) is essential to confirm the diagnosis and could potentially be helpful in predicting the severity of the disease, although further studies are required.*

IVA can be identified by NBS and has been included in NBS programmes in numerous countries [4,50]. Fast processing is necessary to reduce the number of patients who develop symptoms before obtaining the result of NBS. As blood isovalerylcarnitine cannot be differentiated from other 5-carbon unit acyl isomers by direct flow injection mass spectrometry, there is a risk of false positive NBS results. A high number of false-positive NBS results have been documented following maternal intake of pivaloylester-containing antibiotics during the final weeks of pregnancy [43,46,51]. Additionally, elevated pivaloylcarnitine concentrations have been reported in two children following the administration of sivelestat sodium, a neutrophil elastase inhibitor used for the treatment of acute respiratory distress syndrome [52].

Second-tier tests have been implemented to enhance the positive predictive value of an elevated C5-carnitine concentration in NBS. Differentiation of C5 isomers enables screening laboratories to distinguish between false and true positives [53,54,55,56]. After obtaining a positive NBS result, supplementary urine organic acid analysis is necessary to confirm or exclude the diagnosis.

In the initial cohort of patients with mild IVA reported by Ensenauer et al. [12], as well as in a recent analysis of screening results involving 84 patients identified through NBS [19], a combined assessment of C5-carnitine in blood and IVG in urine was proposed as a means of predicting which individuals have the biochemically mild form of the condition. The proposed cutoffs were C5-carnitine < 6 µmol/L plus IVG < 195 mmol/mol creatinine [12] and C5-carnitine < 5.6 µmol/L plus C5-/C0-carnitine ratio < 0.624 or C5-carnitine < 5.6 µmol/L plus IVG < 334 mmol/mol creatinine [19], respectively. The evaluation of larger patient populations is needed and may result in further adjustments of the cutoffs.

#### 3.2.5. Molecular Genetic Analyses and Enzyme Assays

**Statement** **6.** *IVD gene analysis and/or IVD enzyme assay are recommended to confirm the diagnosis of IVA.*

Enzyme assays and metabolite loading tests in cultured skin fibroblasts or leucocytes have been used to confirm the diagnosis of IVA [8].

Given that enzyme assays are only conducted in a limited number of specialized laboratories, sequencing of the *IVD* gene is the preferred method for confirmation in most cases. The correlation between genotype and clinical phenotype is generally poor [17,23]. A definite genotype–phenotype correlation is known for the common variant NM_002225.5:c.932C>T (p.Ala311Val), which has been detected in a homozygous or compound heterozygous state in a high proportion of asymptomatic individuals identified through NBS [12].

Following the identification of disease-causing variants in an index case, it is possible to conduct carrier testing in parents, provide genetic counselling, and perform prenatal diagnosis.

### 3.3. Emergency Treatment

#### 3.3.1. Acute Measures

**Statement** **7.** *In the event of severe metabolic decompensation of an IVA patient, it is critical to initiate therapy immediately.*

The emergency treatment of neonatal crises and subsequent episodes of metabolic decompensation comprises the discontinuation of protein intake, the promotion of anabolism, rehydration, and metabolic drug therapy (Table 2) [17,57].

In the event of severe hyperammonemia, treatment depends on whether the underlying diagnosis has been established. The management of severe hyperammonemia of unknown cause adheres to the published guidelines for the diagnosis and management of urea cycle disorders (UCD) [58,59]. There are no published guidelines for the management of hyperammonemia in patients with confirmed IVA. However, guidelines exist for the emergency treatment of MMA and PA [60]. Hyperammonemia observed in organic acidemias results from inhibition of NAGS, which activates the first enzyme of the urea cycle, carbamoylphosphate synthetase 1 (CPS1) [61,62]. Carbamoylglutamate (carglumic acid) stimulates CPS1, thereby reducing hyperammonemia by inducing the production of carbamoylphosphate, the first by-product of ammonia in the urea cycle. It has been demonstrated that carglumic acid has a beneficial effect on hyperammonemia in MMA and PA [63,64]. Similarly, two retrospective investigations and one case study report a rapid and dramatic decrease in the plasma ammonia concentration in IVA patients treated with carglumic acid [65,66,67]. A persistent tendency to hyperammonemia is less common in patients with IVA than in patients with MMA or PA, and hyperammonemia tends to resolve with correction of metabolic status and usually requires no additional specific treatment. To date, no long-term treatment with carglumic acid has been reported in IVA patients [68].

According to existing guidelines, extracorporeal detoxification therapy is considered in acute hyperammonemic decompensation, if the blood ammonia concentration is within the range of 250 to 500 μmol/L, and started immediately when ammonia concentrations are above 500 µmol/L [60,69]. Different modalities can be applied, including continuous venovenous hemodiafiltration, which is considered the method of choice for the treatment of hyperammonemia [70,71]. Nevertheless, outcome depends on both the severity and the duration of hyperammonemia rather than on the specific dialysis technique employed [72].

Parenteral glucose administration is required to provide a high-energy supply, which is necessary to promote anabolism. Administration of insulin may be considered due to its anabolic effect, but requires close monitoring [57]. The glucose demand is dependent on the age of the child [73]: 8 to 10 mg/kg·min of glucose are recommended for neonates and infants [74]. Oral intake or nasogastric tube feeding of protein-free formula should be started as early as possible. Once a β-oxidation defect has been excluded in a symptomatic neonate, intravenous lipids at 2 g/kg·d can be introduced to further promote anabolism as recommended for MMA and PA patients [60]. After 24 (up to a maximum of 48) hours of emergency treatment, protein is reintroduced successively while monitoring ammonia concentrations and blood gases [60].

L-carnitine is administered to prevent secondary carnitine depletion resulting from isovalerylcarnitine excretion [75]. Intravenous administration of 100 mg/kg·d is recommended for the emergency treatment of MMA and PA [60]; the same treatment is employed for patients with IVA [57,74]. The dose has sometimes been increased to enhance excretion of isovalerylcarnitine, but studies demonstrating efficacy of this approach are lacking.

Glycine supplementation promotes the formation of IVG, a non-toxic conjugate of isovaleric acid, which is rapidly excreted in the urine [76]. Consequently, glycine is frequently included in the long-term treatment of IVA. A reduction in plasma isovaleric acid concentrations and clinical improvement following an increase in oral L-glycine dose during acute metabolic decompensation have been described in individual patients [76,77,78,79]. A comprehensive evaluation of the efficacy and optimal dosage of L-glycine has yet to be conducted.

#### 3.3.2. Laboratory Tests to Monitor Emergency Treatment

**Statement** **8.** *Blood ammonia and lactate concentrations and blood gases should be measured to guide acute treatment.*

Laboratory tests that are useful for the assessment of the severity of a metabolic crisis and guidance of therapy include measurement of ammonia, blood gases, anion gap, lactate, glucose, blood or urine ketones, electrolytes, creatinine, urea, and blood cell count [15,80]. Specific metabolic tests (organic acids, acylcarnitines) are not helpful for monitoring acute therapy.

#### 3.3.3. Sick Day Management at Home

**Statement** **9.** *In case of minor illnesses, a sick day regimen with increased caloric intake and decreased protein intake can be initiated at home. If symptoms persist or worsen, the patient must be admitted to the hospital. In young children, early presentation to the hospital always needs to be considered irrespective of the clinical condition.*

Emergency regimens for sick day management at home comprise anabolization and low protein intake [17,57,74], most effectively achieved by using oral solutions containing glucose polymers and/or leucine-free formula. Similar recommendations have been made for patients with MMA and PA [81].

#### 3.3.4. Perioperative Management

**Statement** **10.** *Peri-interventional fasting must be bridged with low protein parenteral nutrition to prevent metabolic decompensation.*

The MMA/PA guideline provides comprehensive guidance on perioperative management, and these recommendations are also applied for IVA patients in clinical practice [81]. In patients undergoing surgery, it is essential to ensure adequate parenteral caloric intake to prevent catabolism [82]. Following consultation with the anesthetist, food intake is suspended as late as possible. Clear solutions containing glucose polymers can be administered up to 2 h or even less before anesthesia [83]. During periods of fasting, glucose infusion is provided to cover the age-appropriate glucose requirement. For extended procedures or if delayed postoperative food intake is anticipated, the administration of additional intravenous lipids is reasonable (2 g/kg·d). Following a fasting period of 12 to 24 h, intravenous amino acids are administered. L-carnitine is administered intravenously at a dosage of 100 mg/kg·d.

It is recommended that enteral nutrition is resumed as soon as possible following surgery. The infusion therapy is continued until the patients tolerate their usual diet.

### 3.4. Long-Term Treatment

Management of patients with symptomatic forms and those with biochemically mild forms of IVA focusing on long-term strategies are presented separately.

#### 3.4.1. Long-Term Management of Symptomatic IVA

The objective of long-term management is to reduce the production of disease-specific metabolites and enhance their excretion by adjusting protein intake, energy intake, and pharmacotherapy.

##### Protein Intake

**Statement** **11.** *Natural protein intake should be restricted to reduce the isovaleric acid burden while ensuring that the minimum amount of protein intake recommended by FAO/WHO/UNU 2007 and EFSA 2012 is achieved. The supplementation with leucine-free L-amino acid supplements appears useful when the natural protein tolerance is below the minimum required level of protein intake.*

Leucine is an essential amino acid that plays a pivotal role in regulating metabolism by stimulating protein synthesis [84,85]. Over-restriction of leucine can result in weight loss and muscle weakness [86]. In IVA, the restriction of leucine intake results in a reduction of produced toxic metabolites from leucine degradation. At the same time, protein intake must be sufficient to prevent protein catabolism leading to metabolic instability and permit growth [36]. The dietary therapy prescribed for IVA patients varies considerably depending on the severity of the disease and local practice [87].

Natural protein intake should be maintained at a concentration that meets or exceeds the minimum amount of protein intake recommended by the FAO/WHO/UNU 2007 and EFSA 2012 guidelines [88,89] (Table 3). Patients tolerating considerably larger quantities of protein than FAO/WHO/UNU safe levels have been documented [87]. In many cases, dietary restrictions are eased during late childhood or adolescence to allow for an increase in natural protein intake, without any adverse effects on the patient’s condition [18].

The addition of leucine-free L-amino acid supplements is considered in cases where the natural protein or leucine tolerance does not allow sufficient protein or leucine intake for normal growth and health [87]. The quantity of leucine-free L-amino acid supplementation must be tailored on an individual basis, bearing in mind that the use of amino acid supplements has not been demonstrated to be either necessary or efficacious in IVA patients.

The calculation of leucine intake, rather than of protein intake, is not standard practice. However, it has been employed in very unstable patients [87]. The minimum required intake of leucine has been defined (Table 4) [88,89,90].

##### Energy Intake

Energy intake for patients with IVA, as for patients with other organic acidurias, should align with the recommendations set forth by the FAO/WHO/UNU [91], with adjustments made on an individual basis. In the event of an impending catabolic crisis, emergency plans should aim to increase caloric intake compared to the patient’s usual diet [92].

##### Pharmacotherapy

**Statement** **12.** *L-carnitine is recommended for the long-term treatment of IVA, as is the case for other organic acidurias. The dosage should be individually adjusted to maintain a normal concentration of free carnitine in blood. Patients with metabolically severe types of IVA are additionally treated with L-glycine. There are no studies confirming the clinical effect of this therapy.*

L-carnitine can be conjugated with isovaleryl-CoA to form non-toxic isovalerylcarnitine, which is efficiently excreted in urine, as an effective physiological detoxification pathway [75]. However, the formation of isovalerylcarnitine can result in a secondary deficiency of carnitine in patients with IVA [93,94], resulting in the need for L-carnitine supplementation. L-carnitine supplementation is well tolerated, and adverse effects are rare (Table S3). However, dose-dependent diarrhea has been reported [95]. Prolonged use at a high dose may result in the development of a fish-like body odor [96]. The need for riboflavin supplementation to treat an unpleasant body odor during L-carnitine therapy has not been reported in patients with IVA.

Recommended doses of L-carnitine vary widely, from 10 to 300 mg/kg·d (Table S3), with a median dosage of 100 mg/kg·d [17,28,92] in two to three daily doses. Often, significantly lower doses than 100 mg/kg·d are sufficient.

The conjugation with glycine to form IVG reduces the accumulation of isovaleric acid in blood [48,78,97]. This reaction can be enhanced through the oral administration of L-glycine.

In individual cases, the administration of L-glycine has been associated with a reduction in the duration and severity of disease symptoms [48,76,97]. Although detailed data are lacking, fewer patients were treated with L-glycine than with L-carnitine in larger patient cohorts [28,29]. The administration of L-glycine is considered in patients with a more severe clinical course. Efficacy and dose-finding studies are lacking, and recommendations on L-glycine dosage are largely derived from case reports. In infants, oral L-glycine doses of 10 to 500 mg/kg·d have been employed (Table S3), and frequently used doses are 150 to 250 mg/kg·d. Generally, L-glycine supplementation is well tolerated. Encephalopathic side-effects including lethargy and ataxia may occur following the administration of exceedingly high doses of L-glycine (300 mg/kg·d), leading to massively elevated plasma concentrations of above 1000 µmol/L [98,99].

#### 3.4.2. Monitoring of Long-Term Therapy

**Statement** **13.** *Regular monitoring of plasma amino acid and free carnitine concentrations in blood is recommended to detect deficiencies in essential amino acids and to adjust the L-carnitine dose.*

Treated patients with IVA require life-long care from both a metabolic specialist and a dietician. Laboratory investigations comprise plasma amino acid analyses every 3 to 12 months depending on the age of the patient [87] to assess possible imbalances or deficiencies of essential amino acids. Expert nutritional counselling is necessary to avoid malnutrition due to insufficient nutrient intake or to prevent excessive intakes. Concentrations of essential amino acids in the normal range should be aimed for.

The free plasma carnitine concentration can guide L-carnitine therapy. L-carnitine supplementation has been demonstrated to increase urinary isovalerylcarnitine concentrations, and the combination with L-glycine therapy, through the conversion of free isovaleric acid into non-toxic carnitine and glycine conjugates excreted in the urine, appears most effective [100]. However, the measurement of isovaleric acid in urine is not routinely used to monitor therapy.

In any patient with a medically modified diet, it is reasonable to undertake regular measurements of vitamins and trace elements. In an international survey on nutritional practices in IVA, the monitoring of zinc, selenium, hemoglobin, ferritin, and vitamins B12, D, A, and E was considered useful [87].

#### 3.4.3. Long-Term Management of Biochemically Mild IVA

**Statement** **14.** *Asymptomatic patients with only mild biochemical abnormalities do not have to adhere to a strict protein-restricted diet.*

Patients with biochemically mild IVA have been asymptomatic, irrespective of the type of treatment [12,18,19]. A strict protein-balanced diet is an unnecessary intervention. The efficacy of L-carnitine supplementation has not been proven in this patient group [17]. It is unlikely that negative effects related to a catabolic state will occur [12]. Patients may consider an emergency regimen during periods of severe catabolic stress [4,18], but refrain from any other specific therapy.

Currently available data suggest that a significant number of patients with mild courses are subjected to excessive treatment [12,18], with potential consequences for patients and families.

### 3.5. Disease Outcome

**Statement** **15.** *As psychomotor development cannot be accurately predicted, symptomatic patients should be connected with a multidisciplinary team comprising pediatric neurologists, physiotherapists, educational therapists, speech therapists, and child psychologists.*

Disease progression with visceral organ manifestation such as chronic renal failure or cardiomyopathy has been reported only infrequently in patients with IVA in contrast to MMA or PA patients [15,18,36]. Optic nerve atrophy has been described in a single patient [101]. Currently available evidence does not indicate the necessity of organ-specific follow-up in the absence of symptoms.

The neurocognitive outcome in IVA varies and ranges from normal development to severe intellectual disability. Severe intellectual impairment and focal neurological symptoms are less prevalent in IVA than in MMA and PA patients [29,102]. The IQ of all published patients with biochemically mild IVA was within the normal range [12,18].

Grünert et al. studied the neurocognitive outcome of 20 patients and supplemented this dataset with data from 108 patients for whom neurocognitive outcomes were available in the literature [28]. A significant inverse correlation was observed between the age at diagnosis and the IQ score in patients who had not experienced more than one severe catabolic episode. More than 80% of early-diagnosed patients, either within the study population or in the literature, exhibited a normal cognitive outcome compared to the group of late-diagnosed patients, where the frequency was slightly below 50%.

In a German cohort comprising 24 patients with classic IVA diagnosed by NBS, individuals who experienced severe neonatal decompensation had significantly lower IQ scores compared to those who did not [18].

A trend towards normal achievement of motor milestones was observed in the screened IVA patients included in the European Registry and Network for Intoxication-Type Metabolic Diseases (E-IMD) as compared to clinically diagnosed patients [29]. However, after the exclusion of patients exhibiting only mild symptoms, this trend became undetectable [29].

As psychomotor development cannot be accurately predicted, symptomatic patients should be connected to a multidisciplinary team comprising pediatric neurologists, physiotherapists, educational therapists, speech therapists, and child psychologists. This approach allows to identify additional support needs and provides comprehensive care for patients.

The administration of standardized developmental assessments to preschool-age children diagnosed with IVA is an effective method for informing decisions regarding the most suitable educational environment.

## 4. Conclusions

This is the first comprehensive systematic search and review of the literature since IVA was described approximately 60 years ago, resulting in 15 statements. Expert opinion and clinical, biochemical, and dietary expertise were included in the literature review and evaluation of data. As evidence-based recommendations for the management of IVA patients are lacking, these statements can form a basis for standardized treatment and follow-up care. In the future, IVA guidelines can be formulated using larger sets of patient data. Undoubtedly, new knowledge from structured long-term follow-up of patients will emerge.

## Figures and Tables

**Table 1:** Baseline laboratory tests in a patient with suspected IVA.

Baseline Laboratory Tests
Metabolic acidosis with elevated anion gap
Elevated ketone bodies (particularly in newborns)
Elevated lactate concentration
Hyperammonemia
Leukopenia, thrombocytopenia, pancytopenia

**Table 2:** Measures of emergency treatment.

Emergency Treatment
Rehydration
Anabolic therapy through intravenous high-energy supply (glucose, insulin, lipids)
Decrease or stop protein intake
Intravenous supplementation of L-carnitine
Oral supplementation of L-glycine
Ammonia scavengers and carglumic acid in hyperammonemia
Extracorporeal detoxification (in case of severe hyperammonemia and insufficient effect of conservative treatment) according to the UCD guideline [58]

**Table 3:** FAO/WHO/UNU 2007 safe levels of protein intake ^†^ [88,89].

Age	1 m	2 m	3 m	6–12 m	1–10 y	11–16 y	>16 y
(g/kg·d)	1.77	1.50	1.36	1.31	0.91–1.14	0.84 (f)–0.91 (m)	0.83 (f)–0.86 (m)

m, month; FAO, Food and Agriculture Organization of the United Nations; UNU, United Nations University; WHO, World Health Organization; y, year; (f), female; (m), male. ^†^ Safe levels of protein intake are defined as the average protein requirement (sum of maintenance and growth requirement) plus 1.96 SD.

**Table 4:** Leucine requirements.

Age Groups	Mean Leucine Requirements FAO/WHO/UNU 2007 [88,89] for the Healthy Population (mg/kg·d)	Leucine for Patients with Disorders of BCAA Metabolism [90] (mg/kg·d)
**0–12 m**	6 m: 73	0–6 m: 65–120 7–12 m: 50–90
**1.0–2.9 y**	54	40–90
**3.0–10.9 y**	44	40–60
**11.0–14.9 y**	44	11.0–12.9 y: 40–60 13.0–14.9 y: 30–60
**15.0–18.0 y**	42	30–60
**>18.0 y**	39	30–60

BCAA, branched-chain amino acids; FAO, Food and Agriculture Organization of the United Nations; m, month; UNU, United Nations University; WHO, World Health Organization; y, year.

## Supplementary Materials

The following supporting information can be downloaded at: https://www.mdpi.com/article/10.3390/ijns11040092/s1, Table S1: Key question checklist for a systematic search and review on isovaleric acidemia (IVA). Table S2: Terms for literature search. Table S3: Long-term treatment with L-carnitine and/or L-glycine.

## References

[1] Tanaka K., Budd M., Efron M., Isselbacher K. (1966). Isovaleric acidemia: A new genetic defect of leucine metabolism. Proc. Natl. Acad. Sci. USA.

[2] Moorthie S., Cameron L., Sagoo G.S., Bonham J.R., Burton H. (2014). Systematic review and meta-analysis to estimate the birth prevalence of five inherited metabolic diseases. J. Inherit. Metab. Dis..

[3] Mütze U., Garbade S.F., Gramer G., Lindner M., Freisinger P., Grünert S.C., Hennermann J., Ensenauer R., Thimm E., Zirnbauer J. (2020). Long-term Outcomes of Individuals With Metabolic Diseases Identified Through Newborn Screening. Pediatrics.

[4] Schlune A., Riederer A., Mayatepek E., Ensenauer R. (2018). Aspects of Newborn Screening in Isovaleric Acidemia. Int. J. Neonatal Screen..

[5] Dionisi-Vici C., Deodato F., Roschinger W., Rhead W., Wilcken B. (2006). ‘Classical’ organic acidurias, propionic aciduria, methylmalonic aciduria and isovaleric aciduria: Long-term outcome and effects of expanded newborn screening using tandem mass spectrometry. J. Inherit. Metab. Dis..

[6] Parimoo B., Tanaka K. (1993). Structural organization of the human isovaleryl-CoA dehydrogenase gene. Genomics.

[7] Ikeda Y., Dabrowski C., Tanaka K. (1983). Separation and properties of five distinct acyl-CoA dehydrogenases from rat liver mitochondria. Identification of a new 2-methyl branched chain acyl-CoA dehydrogenase. J. Biol. Chem..

[8] Rhead W.J., Tanaka K. (1980). Demonstration of a specific mitochondrial isovaleryl-CoA dehydrogenase deficiency in fibroblasts from patients with isovaleric acidemia. Proc. Natl. Acad. Sci. USA.

[9] Ribeiro C.A., Balestro F., Grando V., Wajner M. (2007). Isovaleric acid reduces Na^+^, K^+^-ATPase activity in synaptic membranes from cerebral cortex of young rats. Cell Mol. Neurobiol..

[10] Leipnitz G., Seminotti B., Amaral A.U., de Bortoli G., Solano A., Schuck P.F., Wyse A.T., Wannmacher C.M., Latini A., Wajner M. (2008). Induction of oxidative stress by the metabolites accumulating in 3-methylglutaconic aciduria in cerebral cortex of young rats. Life Sci..

[11] Villani G.R., Gallo G., Scolamiero E., Salvatore F., Ruoppolo M. (2017). “Classical organic acidurias”: Diagnosis and pathogenesis. Clin. Exp. Med..

[12] Ensenauer R., Vockley J., Willard J.M., Huey J.C., Sass J.O., Edland S.D., Burton B.K., Berry S.A., Santer R., Grunert S. (2004). A common mutation is associated with a mild, potentially asymptomatic phenotype in patients with isovaleric acidemia diagnosed by newborn screening. Am. J. Hum. Genet..

[13] Spirer Z., Swirsky-Fein S., Zakut V., Legum C., Bogair N., Charles R., Gil-Av E. (1975). Acute neonatal isovaleric acidemia. A report of two cases. Isr. J. Med. Sci..

[14] Tsai A.C., Lin H.T., Chou M., Bolen J., Zimmerman C., DeMarzo D., Enchautegui-Colon Y. (2022). Compound heterozygote variants: C.848A > G; p.Glu283Gly and c.890C > T; p.Ala297Val, of Isovaleric acid-CoA dehydrogenase (IVD) gene causing severe Isovaleric acidemia with hyperammonemia. Mol. Genet. Metab. Rep..

[15] Kölker S., Garcia-Cazorla A., Valayannopoulos V., Lund A.M., Burlina A.B., Sykut-Cegielska J., Wijburg F.A., Teles E.L., Zeman J., Dionisi-Vici C. (2015). The phenotypic spectrum of organic acidurias and urea cycle disorders. Part 1: The initial presentation. J. Inherit. Metab. Dis..

[16] Feinstein J.A., O’Brien K. (2003). Acute metabolic decompensation in an adult patient with isovaleric acidemia. South. Med. J..

[17] Vockley J., Ensenauer R. (2006). Isovaleric acidemia: New aspects of genetic and phenotypic heterogeneity. Am. J. Med. Genet. Part C Semin. Med. Genet..

[18] Mütze U., Henze L., Gleich F., Lindner M., Grünert S.C., Spiekerkoetter U., Santer R., Blessing H., Thimm E., Ensenauer R. (2021). Newborn screening and disease variants predict neurological outcome in isovaleric aciduria. J. Inherit. Metab. Dis..

[19] Mütze U., Henze L., Schröter J., Gleich F., Lindner M., Grünert S.C., Spiekerkoetter U., Santer R., Thimm E., Ensenauer R. (2023). Isovaleric aciduria identified by newborn screening: Strategies to predict disease severity and stratify treatment. J. Inherit. Metab. Dis..

[20] Couce M.L., Aldamiz-Echevarría L., Bueno M.A., Barros P., Belanger-Quintana A., Blasco J., García-Silva M.T., Márquez-Armenteros A.M., Vitoria I., Vives I. (2017). Genotype and phenotype characterization in a Spanish cohort with isovaleric acidemia. J. Hum. Genet..

[21] Barends M., Pitt J., Morrissy S., Tzanakos N., Boneh A. (2014). Biochemical and molecular characteristics of patients with organic acidaemias and urea cycle disorders identified through newborn screening. Mol. Genet. Metab..

[22] Ensenauer R., Fingerhut R., Maier E.M., Polanetz R., Olgemöller B., Röschinger W., Muntau A.C. (2011). Newborn screening for isovaleric acidemia using tandem mass spectrometry: Data from 1.6 million newborns. Clin. Chem..

[23] Dercksen M., Duran M., Ijlst L., Mienie L.J., Reinecke C.J., Ruiter J.P., Waterham H.R., Wanders R.J. (2012). Clinical variability of isovaleric acidemia in a genetically homogeneous population. J. Inherit. Metab. Dis..

[24] Hertecant J.L., Ben-Rebeh I., Marah M.A., Abbas T., Ayadi L., Ben Salem S., Al-Jasmi F.A., Al-Gazali L., Al-Yahyaee S.A., Ali B.R. (2012). Clinical and molecular analysis of isovaleric acidemia patients in the United Arab Emirates reveals remarkable phenotypes and four novel mutations in the IVD gene. Eur. J. Med. Genet..

[25] Ozgul R.K., Karaca M., Kilic M., Kucuk O., Yucel-Yilmaz D., Unal O., Hismi B., Aliefendioglu D., Sivri S., Tokatli A. (2014). Phenotypic and genotypic spectrum of Turkish patients with isovaleric acidemia. Eur. J. Med. Genet..

[26] Zaki O.K., Priya Doss C.G., Ali S.A., Murad G.G., Elashi S.A., Ebnou M.S.A., Kumar D.T., Khalifa O., Gamal R., El Abd H.S.A. (2017). Genotype-phenotype correlation in patients with isovaleric acidaemia: Comparative structural modelling and computational analysis of novel variants. Hum. Mol. Genet..

[27] Mohsen A.W., Anderson B.D., Volchenboum S.L., Battaile K.P., Tiffany K., Roberts D., Kim J.J., Vockley J. (1998). Characterization of molecular defects in isovaleryl-CoA dehydrogenase in patients with isovaleric acidemia. Biochemistry.

[28] Grünert S.C., Wendel U., Lindner M., Leichsenring M., Schwab K.O., Vockley J., Lehnert W., Ensenauer R. (2012). Clinical and neurocognitive outcome in symptomatic isovaleric acidemia. Orphanet J. Rare Dis..

[29] Heringer J., Valayannopoulos V., Lund A.M., Wijburg F.A., Freisinger P., Baric I., Baumgartner M.R., Burgard P., Burlina A.B., Chapman K.A. (2016). Impact of age at onset and newborn screening on outcome in organic acidurias. J. Inherit. Metab. Dis..

[30] Schulze A., Lindner M., Kohlmuller D., Olgemoller K., Mayatepek E., Hoffmann G.F. (2003). Expanded newborn screening for inborn errors of metabolism by electrospray ionization-tandem mass spectrometry: Results, outcome, and implications. Pediatrics.

[31] Wilcken B., Haas M., Joy P., Wiley V., Bowling F., Carpenter K., Christodoulou J., Cowley D., Ellaway C., Fletcher J. (2009). Expanded newborn screening: Outcome in screened and unscreened patients at age 6 years. Pediatrics.

[32] Lindner M., Gramer G., Haege G., Fang-Hoffmann J., Schwab K.O., Tacke U., Trefz F.K., Mengel E., Wendel U., Leichsenring M. (2011). Efficacy and outcome of expanded newborn screening for metabolic diseases-report of 10 years from South-West Germany. Orphanet J. Rare Dis..

[33] Horster F., Baumgartner M.R., Viardot C., Suormala T., Burgard P., Fowler B., Hoffmann G.F., Garbade S.F., Kolker S., Baumgartner E.R. (2007). Long-term outcome in methylmalonic acidurias is influenced by the underlying defect (mut0, mut-, cblA, cblB). Pediatr. Res..

[34] Grunert S.C., Mullerleile S., de Silva L., Barth M., Walter M., Walter K., Meissner T., Lindner M., Ensenauer R., Santer R. (2012). Propionic acidemia: Neonatal versus selective metabolic screening. J. Inherit. Metab. Dis..

[35] Tanaka K. (1990). Isovaleric acidemia: Personal history, clinical survey and study of the molecular basis. Prog. Clin. Biol. Res..

[36] Martin-Hernandez E., Lee P.J., Micciche A., Grunewald S., Lachmann R.H. (2009). Long-term needs of adult patients with organic acidaemias: Outcome and prognostic factors. J. Inherit. Metab. Dis..

[37] Kimmoun A., Abboud G., Strazeck J., Merten M., Gueant J.L., Feillet F. (2008). Acute decompensation of isovaleric acidemia induced by Graves’ disease. Intensive Care Med..

[38] Saudubray J.M., Ogier H., Bonnefont J.P., Munnich A., Lombes A., Herve F., Mitchel G., The B.P., Specola N., Parvy P. (1989). Clinical approach to inherited metabolic diseases in the neonatal period: A 20-year survey. J. Inherit. Metab. Dis..

[39] Attia N., Sakati N., al Ashwal A., al Saif R., Rashed M., Ozand P.T. (1996). Isovaleric acidemia appearing as diabetic ketoacidosis. J. Inherit. Metab. Dis..

[40] Kahler S.G., Sherwood W.G., Woolf D., Lawless S.T., Zaritsky A., Bonham J., Taylor C.J., Clarke J.T., Durie P., Leonard J.V. (1994). Pancreatitis in patients with organic acidemias. J. Pediatr..

[41] Ho G., Yonezawa A., Masuda S., Inui K., Sim K.G., Carpenter K., Olsen R.K., Mitchell J.J., Rhead W.J., Peters G. (2011). Maternal riboflavin deficiency, resulting in transient neonatal-onset glutaric aciduria Type 2, is caused by a microdeletion in the riboflavin transporter gene GPR172B. Hum. Mutat..

[42] Rolland M.O., Divry P., Zabot M.T., Guibaud P., Gomez S., Lachaux A., Loras I. (1991). Isolated 3-methylcrotonyl-CoA carboxylase deficiency in a 16-month-old child. J. Inherit. Metab. Dis..

[43] Abdenur J.E., Chamoles N.A., Guinle A.E., Schenone A.B., Fuertes A.N. (1998). Diagnosis of isovaleric acidaemia by tandem mass spectrometry: False positive result due to pivaloylcarnitine in a newborn screening programme. J. Inherit. Metab. Dis..

[44] Gibson K.M., Burlingame T.G., Hogema B., Jakobs C., Schutgens R.B., Millington D., Roe C.R., Roe D.S., Sweetman L., Steiner R.D. (2000). 2-Methylbutyryl-coenzyme A dehydrogenase deficiency: A new inborn error of L-isoleucine metabolism. Pediatr. Res..

[45] Andresen B.S., Christensen E., Corydon T.J., Bross P., Pilgaard B., Wanders R.J., Ruiter J.P., Simonsen H., Winter V., Knudsen I. (2000). Isolated 2-methylbutyrylglycinuria caused by short/branched-chain acyl-CoA dehydrogenase deficiency: Identification of a new enzyme defect, resolution of its molecular basis, and evidence for distinct acyl-CoA dehydrogenases in isoleucine and valine metabolism. Am. J. Hum. Genet..

[46] Bonham J.R., Carling R.S., Lindner M., Franzson L., Zetterstrom R., Boemer F., Cerone R., Eyskens F., Vilarinho L., Hougaard D.M. (2018). Raising Awareness of False Positive Newborn Screening Results Arising from Pivalate-Containing Creams and Antibiotics in Europe When Screening for Isovaleric Acidaemia. Int. J. Neonatal Screen..

[47] Boemer F., Schoos R., de Halleux V., Kalenga M., Debray F.G. (2014). Surprising causes of C5-carnitine false positive results in newborn screening. Mol. Genet. Metab..

[48] Krieger I., Tanaka K. (1976). Therapeutic effects of glycine in isovaleric acidemia. Pediatr. Res..

[49] Ando T., Klingberg W., Ward A., Rasmussen K., Nyhan W. (1971). Isovaleric acidemia presenting with altered metabolism of glycine. Pediatr. Res..

[50] Therrell B.L., Padilla C.D., Borrajo G.J.C., Khneisser I., Schielen P.C.J.I., Knight-Madden J., Malherbe H.L., Kase M. (2024). Current Status of Newborn Bloodspot Screening Worldwide 2024: A Comprehensive Review of Recent Activities (2020–2023). Int. J. Neonatal Screen..

[51] Fernández M.R., Besga B.G., Montero A.P., Dulín E.Í. (2018). C5-carnitin increase in newborns due to pre-labour treatment with cefditoren pivoxil. Rev. Esp. Salud Publica.

[52] Yamada K., Kobayashi H., Bo R., Takahashi T., Hasegawa Y., Nakamura M., Ishige N., Yamaguchi S. (2015). Elevation of pivaloylcarnitine by sivelestat sodium in two children. Mol. Genet. Metab..

[53] Carling R.S., Burden D., Hutton I., Randle R., John K., Bonham J.R. (2018). Introduction of a Simple Second Tier Screening Test for C5 Isobars in Dried Blood Spots: Reducing the False Positive Rate for Isovaleric Acidaemia in Expanded Newborn Screening. JIMD Rep..

[54] Minkler P.E., Stoll M.S.K., Ingalls S.T., Hoppel C.L. (2017). Selective and accurate C5 acylcarnitine quantitation by UHPLC-MS/MS: Distinguishing true isovaleric acidemia from pivalate derived interference. J. Chromatogr. B Anal. Technol. Biomed. Life Sci..

[55] Murko S., Aseman A.D., Reinhardt F., Gramer G., Okun J.G., Mütze U., Santer R. (2023). Neonatal screening for isovaleric aciduria: Reducing the increasingly high false-positive rate in Germany. JIMD Rep..

[56] Forni S., Fu X., Palmer S.E., Sweetman L. (2010). Rapid determination of C4-acylcarnitine and C5-acylcarnitine isomers in plasma and dried blood spots by UPLC-MS/MS as a second tier test following flow-injection MS/MS acylcarnitine profile analysis. Mol. Genet. Metab..

[57] Ogier de Baulny H. (2002). Management and emergency treatments of neonates with a suspicion of inborn errors of metabolism. Semin. Neonatol. SN.

[58] Haberle J., Burlina A., Chakrapani A., Dixon M., Karall D., Lindner M., Mandel H., Martinelli D., Pintos-Morell G., Santer R. (2019). Suggested guidelines for the diagnosis and management of urea cycle disorders: First revision. J. Inherit. Metab. Dis..

[59] Savy N., Brossier D., Brunel-Guitton C., Ducharme-Crevier L., Du Pont-Thibodeau G., Jouvet P. (2018). Acute pediatric hyperammonemia: Current diagnosis and management strategies. Hepat. Med..

[60] Baumgartner M.R., Horster F., Dionisi-Vici C., Haliloglu G., Karall D., Chapman K.A., Huemer M., Hochuli M., Assoun M., Ballhausen D. (2014). Proposed guidelines for the diagnosis and management of methylmalonic and propionic acidemia. Orphanet J. Rare Dis..

[61] Coude F.X., Grimber G., Parvy P., Rabier D. (1983). Role of N-acetylglutamate and acetyl-CoA in the inhibition of ureagenesis by isovaleric acid in isolated rat hepatocytes. Biochim. Biophys. Acta.

[62] Häberle J. (2013). Clinical and biochemical aspects of primary and secondary hyperammonemic disorders. Arch. Biochem. Biophys..

[63] Gebhardt B., Vlaho S., Fischer D., Sewell A., Böhles H. (2003). N-carbamylglutamate enhances ammonia detoxification in a patient with decompensated methylmalonic aciduria. Mol. Genet. Metab..

[64] Jones S., Reed C.A., Vijay S., Walter J.H., Morris A.A. (2008). N-carbamylglutamate for neonatal hyperammonaemia in propionic acidaemia. J. Inherit. Metab. Dis..

[65] Chakrapani A., Valayannopoulos V., Segarra N.G., Del Toro M., Donati M.A., García-Cazorla A., González M.J., Plisson C., Giordano V. (2018). Effect of carglumic acid with or without ammonia scavengers on hyperammonaemia in acute decompensation episodes of organic acidurias. Orphanet J. Rare Dis..

[66] Valayannopoulos V., Baruteau J., Delgado M.B., Cano A., Couce M.L., Del Toro M., Donati M.A., Garcia-Cazorla A., Gil-Ortega D., Gomez-de Quero P. (2016). Carglumic acid enhances rapid ammonia detoxification in classical organic acidurias with a favourable risk-benefit profile: A retrospective observational study. Orphanet J. Rare Dis..

[67] Kasapkara C.S., Ezgu F.S., Okur I., Tumer L., Biberoglu G., Hasanoglu A. (2011). N-carbamylglutamate treatment for acute neonatal hyperammonemia in isovaleric acidemia. Eur. J. Pediatr..

[68] Burlina A., Bettocchi I., Biasucci G., Bordugo A., Gasperini S., La Spina L., Maines E., Meli C., Menni F., Paci S. (2022). Long-term use of carglumic acid in methylmalonic aciduria, propionic aciduria and isovaleric aciduria in Italy: A qualitative survey. Eur. Rev. Med. Pharmacol. Sci..

[69] Häberle J. (2011). Clinical practice: The management of hyperammonemia. Eur. J. Pediatr..

[70] Picca S., Dionisi-Vici C., Bartuli A., De Palo T., Papadia F., Montini G., Materassi M., Donati M.A., Verrina E., Schiaffino M.C. (2015). Short-term survival of hyperammonemic neonates treated with dialysis. Pediatr. Nephrol..

[71] Arbeiter A.K., Kranz B., Wingen A.M., Bonzel K.E., Dohna-Schwake C., Hanssler L., Neudorf U., Hoyer P.F., Büscher R. (2010). Continuous venovenous haemodialysis (CVVHD) and continuous peritoneal dialysis (CPD) in the acute management of 21 children with inborn errors of metabolism. Nephrol. Dial. Transplant..

[72] Celik M., Akdeniz O., Ozgun N., Ipek M.S., Ozbek M.N. (2019). Short-term results of continuous venovenous haemodiafiltration versus peritoneal dialysis in 40 neonates with inborn errors of metabolism. Eur. J. Pediatr..

[73] Huidekoper H.H., Ackermans M.T., Ruiter A.F., Sauerwein H.P., Wijburg F.A. (2014). Endogenous glucose production from infancy to adulthood: A non-linear regression model. Arch. Dis. Child..

[74] Prietsch V., Lindner M., Zschocke J., Nyhan W.L., Hoffmann G.F. (2002). Emergency management of inherited metabolic diseases. J. Inherit. Metab. Dis..

[75] Roe C.R., Millington D.S., Maltby D.A., Kahler S.G., Bohan T.P. (1984). L-carnitine therapy in isovaleric acidemia. J. Clin. Invest..

[76] Shigematsu Y., Sudo M., Momoi T., Inoue Y., Suzuki Y., Kameyama J. (1982). Changing plasma and urinary organic acid levels in a patient with isovaleric acidemia during an attack. Pediatr. Res..

[77] Velazquez A., Prieto E.C. (1980). Glycine in acute management of isovalericacidaemia. Lancet.

[78] Cohn R.M., Yudkoff M., Rothman R., Segal S. (1978). Isovaleric acidemia: Use of glycine therapy in neonates. N. Engl. J. Med..

[79] Berry G.T., Yudkoff M., Segal S. (1988). Isovaleric acidemia: Medical and neurodevelopmental effects of long-term therapy. J. Pediatr..

[80] Häberle J., Chakrapani A., Ah Mew N., Longo N. (2018). Hyperammonaemia in classic organic acidaemias: A review of the literature and two case histories. Orphanet J. Rare Dis..

[81] Forny P., Hörster F., Ballhausen D., Chakrapani A., Chapman K.A., Dionisi-Vici C., Dixon M., Grünert S.C., Grunewald S., Haliloglu G. (2021). Guidelines for the diagnosis and management of methylmalonic acidaemia and propionic acidaemia: First revision. J. Inherit. Metab. Dis..

[82] Ruzkova K., Weingarten T.N., Larson K.J., Friedhoff R.J., Gavrilov D.K., Sprung J. (2015). Anesthesia and organic aciduria: Is the use of lactated Ringer’s solution absolutely contraindicated?. Pediatr. Anaesth..

[83] Thomas M., Morrison C., Newton R., Schindler E. (2018). Consensus statement on clear fluids fasting for elective pediatric general anesthesia. Paediatr. Anaesth..

[84] Anthony T.G., McDaniel B.J., Byerley R.L., McGrath B.C., Cavener D.R., McNurlan M.A., Wek R.C. (2004). Preservation of liver protein synthesis during dietary leucine deprivation occurs at the expense of skeletal muscle mass in mice deleted for eIF2 kinase GCN2. J. Biol. Chem..

[85] Schriever S.C., Deutsch M.J., Adamski J., Roscher A.A., Ensenauer R. (2013). Cellular signaling of amino acids towards mTORC1 activation in impaired human leucine catabolism. J. Nutr. Biochem..

[86] Zhu X., Krasnow S.M., Roth-Carter Q.R., Levasseur P.R., Braun T.P., Grossberg A.J., Marks D.L. (2012). Hypothalamic signaling in anorexia induced by indispensable amino acid deficiency. Am. J. Physiol. Endocrinol. Metab..

[87] Pinto A., Daly A., Evans S., Almeida M.F., Assoun M., Belanger-Quintana A., Bernabei S., Bollhalder S., Cassiman D., Champion H. (2017). Dietary practices in isovaleric acidemia: A European survey. Mol. Genet. Metab. Rep..

[88] WHO (2007). Protein and Amino Acid Requirements in Human Nutrition.

[89] EFSA Panel on Dietetic Products Nutrition Allergies (2012). Scientific Opinion on Dietary Reference Values for protein. EFSA J..

[90] Marriage B., Acosta P.B. (2010). Nutrition management of patients with inherited disorders of branched-chain amino acid metabolism. Nutrition Management of Patients with Inherited Metabolic Disorders.

[91] FAO/WHO/UNU (2004). Human energy requirements. Report of a Joint FAO/WHO/UNU Expert Consultation. FAO Food and Nutrition Technical Report Series No. 1..

[92] Bodamer O.A., Hoffmann G.F., Visser G.H., Janecke A., Linderkamp O., Leonard J.V., Fasoli L., Rating D. (1997). Assessment of energy expenditure in metabolic disorders. Eur. J. Pediatr..

[93] Chalmers R.A., Roe C.R., Stacey T.E., Hoppel C.L. (1984). Urinary excretion of l-carnitine and acylcarnitines by patients with disorders of organic acid metabolism: Evidence for secondary insufficiency of l-carnitine. Pediatr. Res..

[94] Stanley C.A., Berry G.T., Bennett M.J., Willi S.M., Treem W.R., Hale D.E. (1993). Renal handling of carnitine in secondary carnitine deficiency disorders. Pediatr. Res..

[95] Mayatepek E., Kurczynski T.W., Hoppel C.L. (1991). Long-term L-carnitine treatment in isovaleric acidemia. Pediatr. Neurol..

[96] Rocher F., Caruba C., Broly F., Lebrun C. (2011). L-carnitine treatment and fish odor syndrome: An unwaited adverse effect. Rev. Neurol..

[97] Yudkoff M., Cohn R.M., Puschak R., Rothman R., Segal S. (1978). Glycine therapy in isovaleric acidemia. J. Pediatr..

[98] Chalmers R.A., de Sousa C., Tracey B.M., Stacey T.E., Weaver C., Bradley D. (1985). L-carnitine and glycine therapy in isovaleric acidaemia. J. Inherit. Metab. Dis..

[99] van Vliet D., Derks T.G., van Rijn M., de Groot M.J., MacDonald A., Heiner-Fokkema M.R., van Spronsen F.J. (2014). Single amino acid supplementation in aminoacidopathies: A systematic review. Orphanet J. Rare Dis..

[100] Itoh T., Ito T., Ohba S., Sugiyama N., Mizuguchi K., Yamaguchi S., Kidouchi K. (1996). Effect of carnitine administration on glycine metabolism in patients with isovaleric acidemia: Significance of acetylcarnitine determination to estimate the proper carnitine dose. Tohoku J. Exp. Med..

[101] Kölker S., Valayannopoulos V., Burlina A.B., Sykut-Cegielska J., Wijburg F.A., Teles E.L., Zeman J., Dionisi-Vici C., Barić I., Karall D. (2015). The phenotypic spectrum of organic acidurias and urea cycle disorders. Part 2: The evolving clinical phenotype. J. Inherit. Metab. Dis..

[102] Nizon M., Ottolenghi C., Valayannopoulos V., Arnoux J.B., Barbier V., Habarou F., Desguerre I., Boddaert N., Bonnefont J.P., Acquaviva C. (2013). Long-term neurological outcome of a cohort of 80 patients with classical organic acidurias. Orphanet J. Rare Dis..

