# Supplementary material for: Practical Considerations for the Diagnosis and Management of Isovaleryl-CoA-Dehydrogenase Deficiency (Isovaleric Acidemia): Systematic Search and Review and Expert Opinions
Source: Int J Neonatal Screen. 2025 Oct 10;11(4):92. doi: 10.3390/ijns11040092 (PMC12551068; doi:10.3390/ijns11040092)
Supplement: Supplementary file 1 [file IJNS-11-00092-s001.zip › IJNS-3599666-supplementary.pdf]

Table S1. Key question checklist for a systematic search and review on isovaleric acidemia (IVA)

A) Clinical symptoms, incidence & diagnosis

Clinical diagnosis

1. What were the clinical signs and symptoms that led to the suspicion of IVA?
2. Which trigger factors for metabolic crises/episodes are reported?
3. Did patients identified by newborn screening (NBS) present with any symptoms at the time of diagnosis?
4. Did patients develop any symptoms (acute or chronic) after the diagnosis was made (either by selective screening or NBS)?
5. How often did the respective patients have symptomatic episodes?

Incidence

6. What is the reported incidence of IVA?

Biochemical analysis

7. How was the diagnosis made?
    a. Metabolites: isovalerylglycine, isovalerylcarnitine, other related metabolites (i.e. Krebs cycle metabolites)
    b. Enzyme analysis/flux assay
    c. Mutation analysis

B) Treatment & monitoring

Emergency (sick day hospital)

8. Which emergency treatment regimens were used? Did they change with age?
9. Which laboratory investigations were used to help guiding acute treatment?
10. Was extracorporeal detoxification used?

Sick day home management

11. Which treatment regimens were used? Did they change with age?

Diet (long-term therapy)

12. Was dietary protein restriction part of the long-term treatment of IVA?
13. Which amount of natural (complete) protein for age was used?
14. What was the age-related leucine intake?
15. What was the age-related amount of amino acid supplement?

Medication (long-term therapy)

16. Were carnitine and/or glycine used in managing IVA in the long-term? Did medication change with age?
17. What other therapeutic approaches were used in treating IVA?
18. Which side effects of medication are reported?

Monitoring

19. Which metabolic/biochemical tests were used to monitor therapy (e.g. isovalerylglycine; C5-carnitine in blood, urine)?

C) Outcome

20. What was the long-term outcome of the different forms of IVA (diagnosis by NBS vs. selective screening) in terms of morbidity? (neurocognitive outcome: IQ, developmental delay, behavioral abnormalities, learning disabilities, school delay, type of school; pancreatitis; comorbidities)
21. What was the clinical outcome in prenatally diagnosed patients?
22. Which information has been reported on mortality and cause and age of death?

Table S2. Terms for literature search

Search terms
“isovaleric acidemia”
“isovaleric aciduria”,
“isovaleric acid”
“isovaleryl-CoA dehydrogenase”
“IVD gene”
“isovaleryl glycine”
“isovalerylglycine”
“isovaleryl carnitine”
“isovaleryl-carnitine”
“isovalerylcarnitine”
“newborn screening AND metabolic disease”
“neonatal screening AND metabolic disease”
“newborn screening AND biochemical genetics”
“neonatal screening AND biochemical genetics”
“newborn screening AND inborn error of metabolism”
“neonatal screening AND inborn error of metabolism”
“newborn screening AND inborn errors of metabolism”
“neonatal screening AND inborn errors of metabolism”
“newborn screening AND inherited metabolic disease”
“neonatal screening AND inherited metabolic disease”

Table S3. Long-term treatment with L-carnitine and/or L-glycine

Reference	All patients treated with L-carnitine and/or L-glycine (n)	Age of patients	Patients treated with L-carnitine (n)	L-carnitine dosage	Patients treated with L-glycine (n)	L-glycine dosage	Tolerability of therapy with L-carnitine or L-glycine or combined therapy
Attia et al. 1996 [1]	1	19 m	1	200 mg/kg*d	1	500 mg/kg*d	
Barends et al. 2014 [2]	4		4	Dosage not indicated	3	Dosage not indicated	
Beauvais et al. 1985 [3]	1	newborn	1	Dosage not indicated	1	250 mg/kg*d	
Berry et al. 1984 [4]	7	1-10 y			7	250 mg/kg*d	
Berry et al. 1988 [5]	9	1-14 y	4	50 mg/kg*d	9	250 mg/kg*d; after toddler age: 180 mg/kg*d	
Castelnovi et al. 2010 [6]	1	20 y, pregnant	1	70 mg/kg*d	1	140 mg/kg*d, no change to pre-conceptional regimen	
Castorina et al. 2008 [7]	1				1	250 mg/kg*d	good clinical condition
	1		1	50 mg/kg*d	1	250 mg/kg*d	good clinical condition
Chalmers et al. 1985 [8]	1	2.5 y	1	40 mg /kg*d			
Cohn et al. 1978 [9]	2	newborn			1	800 mg/d in	good clinical condition
Couce et al. 2017 [10]	10	1 m-18 y	10	Dosage not indicated	8	Dosage not indicated	no side effects
Coude et al. 1982 [11]	4				4	Dosage not indicated	
Dercksen et al. 2012 [12]	10		10	20-100 mg/kg*d	8	10-100 mg/kg*d	
Dodelson et al. 1992 [13]	1	1 y			1	250 mg/kg*d	
Duran et al. 1979 [14]	2	2.5 y/ 4.5 y			2	Dosage not indicated	

Duran et al. 1985 [15]	1		1	1 g/d			
Elsas et al.1988 [16]	2	9 y / 24 m			2	2 g/d (chronic)/ 250 mg/kg*d (metabolic decompensation)	
Ensenauer et al. 2003 [17]	22		22		22	Dosage not indicated	
Ensenauer et al. 2004 [18]	19	2 m-5 y	19	15-150 mg/kg*d			
Erdem et al. 2010 [19]	1	10 y	1	100 mg/kg/d			
Feinstein et al.2003 [20]	1	18 y	1	Dosage not indicated	1	Dosage not indicated	
Fries et al. 1996 [21]	1	7 y	1	started at 50 mg/kg*d gradually increased to 100 mg/kg*d			
Gabriel et al. 1984 [22]	1	newborn	1	250 mg/kg*d	1	250 mg/kg*d	
Gilbert-Barness et al. 1999 [23]	1	newborn (10 d)	1	100 mg/kg*d	1	160 mg/kg*d	
Grünert et al. 2012 [24]	20		17	10-144 mg/kg*d (median 100 mg/kg*d)	9	53-200 mg/kg*d (median 112 mg/kg*d)	
Habets et al. 2012 [25]	3		3	up to 10 g/d during pregnancy	3	up to 100 g/day	
Heimler et al. 1988 [26]	1	2 y			1	250 mg/kg/d	good clinical condition
Heringer et al. 2016 [27]	65	Median age 8,3 y	65	Dosage not indicated	37	Dosage not indicated	
Hu et al. 2020 [28]	15			Dosage not indicated		Dosage not indicated	
Ito et al. 1995 [29]	2	4 y/5 y	2	40 mg/kg*d	2	40-50 mg/kg*d	good clinical condition
James et al. 2006 [30]				Dosage not indicated		Dosage not indicated	
Kahler et al. 1994 [31]	3		3	Dosage not indicated			

Kasapkara et al. 2011 [32]	1	newborn (3 d)	1	100 mg/kg*d	1	150 mg/kg*d	
Kimmoun et al. 2008 [33]	1	24 y	1	Dosage not indicated			
Krieger et al. 1976 [34]	1	5,5 y			1	250 mg/kg*d	
Lee et al. 1998 [35]	1	12 y	1	100 mg/kg*d			
Lee et al. 2010 [36]	1	newborn			1	Dosage not indicated	good clinical condition
Li et al. 2024 [37]	1	7 y	1	100 mg/kg*d			
Lin et al. 2007 [38]	6		6	Dosage not indicated	6	Dosage not indicated	
Loots et al. 2007 [39]	6		6	Dosage not indicated	6	Dosage not indicated	
Lund et al. 2011 [40]	4		4	Dosage not indicated	4	Dosage not indicated	
Malbora et al. 2010 [41]	1		1	Dosage not indicated			
Martin-Hernandez et al. 2006 [42]				100-300 mg/kg*d		150-300 mg/kg*d	
Mayatepek et al. 1991 [43]	1	24 m	1	100 mg/kg*d with 5,5 y; 60 mg/kg*d at 8 y			
Minkler et al. 2008 [44]	1		1	Dosage not indicated			
Miyamoto et al. 2022 [45]	1	18 y	1	500 mg/d	1	6,5 g/d	
Ogier et al. 2002 [46]		newborn		50-100 mg/kg*d		150-300 mg/kg*d	
Pascarella et al. 2011 [47]	2		2	Dosage not indicated	2	Dosage not indicated	good clinical condition
Pesce et al. 1991 [48]	1		1		1	300 mg/kg*d	
Pinto et al. 2017 [49]	133		97%	Dosage not indicated	76%	Dosage not indicated	
Poorthuis et al. 1993 [50]	1		1	30 mg/kg*d			
Rabier et al. 1992 [51]	3		3	250 mg/kg*d	3	100-200 mg/kg*d	
Rakheja et al. 2005 [52]	1	newborn	1	100 mg/kg*d	1	270 mg/kg*d	good clinical condition

Ravikumar et al. 2024 [53]	1	Mid-30s		Dosage not indicated			
Roe et al. 1984 [54]	1	4 y	1	100 mg/kg*d			
Sahai et al. 2011 [55]	1	3 y	1	Dosage not indicated			
Salamino et al. 1994 [56]	1	12 y	1	200 mg/kg*d	1	250 mg/kg/d	
Sogut et al. 2004 [57]	1	19 m	1	Dosage not indicated			
Szymanska et al. 2020 [58]	10	Up to 32 y	9	Dosage not indicated	6	Dosage not indicated	
Tajima et al. 2005 [59]	1	14 y	1	Dosage not indicated			
Tan et al. 2016 [60]	1	9 d	1				
Tokatli et al. 1998 [61]	1	7 y/ 5.5 y	1	Dosage not indicated	1	Dosage not indicated	
	2	10 y,	2	100 mg/kg*d	2		
Truscott et al. 1981 [62]	1	newborn			1	2g/d	
Tsai et al. 2022 [63]	1	newborn		Dosage not indicated			
van Hove et al. 1994 [64]	1	2.5 y	1	100 mg/kg*d			good clinical condition
Vockley et al. 2006 [65]				100 mg/kg*d in classic patients; 30-50 mg/kg*d in mild patients		150-250 mg/kg*d in classic patients	
Wasant et al. 2008 [66]	2		2	Dosage not indicated	2	Dosage not indicated	
Wei et al. 2004 [67]	1		1	Dosage not indicated	1	Dosage not indicated	
Weinberg et al. 1997 [68]	1	16 y	1	Dosage not indicated	1	Dosage not indicated	good clinical condition
Wilson et al. 1984 [69]	1	newborn			1	250 mg/kg*d	
Yudkoff et al. 1978 [70]	1	3.5 y			1	2 g/d	
Zaki et al. 2017 [71]	8	1.5-8 y	6	Dosage not indicated	4	Dosage not indicated	Recurrent vomiting
Zegarra et al. 2023 [72]	1	3 y	1	1500 mg/d			

d, days; m, months; n, number; y, years

References

1. Attia, N.; Sakati, N.; al Ashwal, A.; al Saif, R.; Rashed, M.; et al. Isovaleric acidemia appearing as diabetic ketoacidosis. *J Inherit Metab Dis* **1996**, *19*, 85-86, doi:10.1007/bf01799353.
2. Barends, M.; Pitt, J.; Morrissy, S.; Tzanakos, N.; Boneh, A. Biochemical and molecular characteristics of patients with organic acidaemias and urea cycle disorders identified through newborn screening. *Mol Genet Metab* **2014**, *113*, 46-52, doi:10.1016/j.ymgme.2014.07.003.
3. Beauvais, P.; Peter, M.O.; Barbier, B. [Neonatal form of isovaleric acidemia. Apropos of a new case]. *Arch Fr Pediatr* **1985**, *42*, 531-533.
4. Berry, G.; Yudkoff, M.; Segal, S. Effect of glycine therapy on developmental outcome in isovaleric acidemia. *Pediatr Res* **1984**, *18*, 290A-290A, doi:10.1203/00006450-198404001-01184.
5. Berry, G.T.; Yudkoff, M.; Segal, S. Isovaleric acidemia: medical and neurodevelopmental effects of long-term therapy. *J Pediatr* **1988**, *113*, 58-64, doi:10.1016/s0022-3476(88)80528-6.
6. Castelnovi, C.; Moseley, K.; Yano, S. Maternal isovaleric acidemia: observation of distinctive changes in plasma amino acids and carnitine profiles during pregnancy. *Clin Chim Acta* **2010**, *411*, 2101-2103, doi:10.1016/j.cca.2010.08.023.
7. Castorina, M.; Rigante, D.; Antuzzi, D.; Sciascia Cannizzaro, G.; Ricci, R. Different outcome in isovaleric acidemia might be related to unsatisfactory diet compliance. *Scand J Gastroenterol* **2008**, *43*, 767-768, doi:10.1080/00365520801912128.
8. Chalmers, R.A.; de Sousa, C.; Tracey, B.M.; Stacey, T.E.; Weaver, C.; et al. L-carnitine and glycine therapy in isovaleric acidaemia. *J Inherit Metab Dis* **1985**, *8 Suppl 2*, 141-142, doi:10.1007/bf01811499.
9. Cohn, R.M.; Yudkoff, M.; Rothman, R.; Segal, S. Isovaleric acidemia: use of glycine therapy in neonates. *N Engl J Med* **1978**, *299*, 996-999, doi:10.1056/nejm197811022991807.
10. Couce, M.L.; Aldamiz-Echevarría, L.; Bueno, M.A.; Barros, P.; Belanger-Quintana, A.; et al. Genotype and phenotype characterization in a Spanish cohort with isovaleric acidemia. *J Hum Genet* **2017**, *62*, 355-360, doi:10.1038/jhg.2016.144.
11. Coude, F.X.; Ogier, H.; Grimber, G.; Parvy, P.; Pham Dinh, D.; et al. Correlation between blood ammonia concentration and organic acid accumulation in isovaleric and propionic acidemia. *Pediatrics* **1982**, *69*, 115-117.
12. Dercksen, M.; Duran, M.; Ijlst, L.; Mienie, L.J.; Reinecke, C.J.; et al. Clinical variability of isovaleric acidemia in a genetically homogeneous population. *J Inherit Metab Dis* **2012**, *35*, 1021-1029, doi:10.1007/s10545-012-9457-2.
13. Dodelson de Kremer, R.; Depetris de Boldini, C.; Paschini de Capra, A.; Hliba, E.; Corbella, L. [Phenotypic expression variation of isovaleric acidemia in Argentinian patients. A long term follow-up]. *Medicina (B Aires)* **1992**, *52*, 131-140.
14. Duran, M.; van Sprang, F.J.; Drewes, J.G.; Bruinvis, L.; Ketting, D.; et al. Two sisters with isovaleric acidaemia, multiple attacks of ketoacidosis and normal development. *Eur J Pediatr* **1979**, *131*, 205-211, doi:10.1007/bf00538944.
15. Duran, M.; Dorland, L.; Wadman, S.K. Urinary acylcarnitines in various organic acidemias analyzed by mass spectrometry and h-nuclear magnetic resonance spectroscope. *Pediatr Res* **1985**, *19*, 1086-1086, doi:10.1203/00006450-198510000-00108.
16. Elsas, L.J., 2nd; Naglak, M. Acute and chronic-intermittent isovaleric acidemia: diagnosis and glycine therapy. *Acta Paediatr Jpn* **1988**, *30*, 442-451, doi:10.1111/j.1442-200x.1988.tb02535.x.
17. Ensenauer, R.; Gruenert, S.; Willard, J.; Matern, D.; Wendel, U.; et al. Natural history of isovaleric acidemia (IVA). *J Inherit Metab Dis* **2003**, *26*, 38, doi:https://doi.org/10.1023/A:1024806611687.

18. Ensenauer, R.; Vockley, J.; Willard, J.M.; Huey, J.C.; Sass, J.O.; et al. A common mutation is associated with a mild, potentially asymptomatic phenotype in patients with isovaleric acidemia diagnosed by newborn screening. *Am J Hum Genet* **2004**, *75*, 1136-1142, doi:10.1086/426318.
19. Erdem, E.; Cayonu, N.; Uysalol, E.; Yildirmak, Z.Y. Chronic intermittent form of isovaleric acidemia mimicking diabetic ketoacidosis. *J Pediatr Endocrinol Metab* **2010**, *23*, 503-505, doi:10.1515/jpem.2010.082.
20. Feinstein, J.A.; O'Brien, K. Acute metabolic decompensation in an adult patient with isovaleric acidemia. *South Med J* **2003**, *96*, 500-503, doi:10.1097/01.Smj.0000051141.03668.1d.
21. Fries, M.H.; Rinaldo, P.; Schmidt-Sommerfeld, E.; Jurecki, E.; Packman, S. Isovaleric acidemia: response to a leucine load after three weeks of supplementation with glycine, L-carnitine, and combined glycine-carnitine therapy. *J Pediatr* **1996**, *129*, 449-452, doi:10.1016/s0022-3476(96)70081-1.
22. Gabriel, M.; Behbehant, A.W.; Hunneman, D.H. Comparison of glycine and carnitine effect in neonatal isovaleric acidemia. *Pediatric Research* **1984**, *18*, 806-806, doi:10.1203/00006450-198408000-00088.
23. Gilbert-Barness, E.; Barness, L.A. Isovaleric acidemia with promyelocytic myeloproliferative syndrome. *Pediatr Dev Pathol* **1999**, *2*, 286-291, doi:10.1007/s100249900125.
24. Grünert, S.C.; Wendel, U.; Lindner, M.; Leichsenring, M.; Schwab, K.O.; et al. Clinical and neurocognitive outcome in symptomatic isovaleric acidemia. *Orphanet J Rare Dis* **2012**, *7*, 9, doi:10.1186/1750-1172-7-9.
25. Habets, D.D.; Schaper, N.C.; Rogozinski, H.; van Spronsen, F.J.; van Rijn, M.; et al. Biochemical Monitoring and Management During Pregnancy in Patients with Isovaleric Acidaemia is Helpful to Prevent Metabolic Decompensation. *JIMD Rep* **2012**, *3*, 83-89, doi:10.1007/8904_2011_66.
26. Heimler, R.; Hennes, H.; Khayata, S.; Sasidharan, P.; Matalon, R. Isovaleric acidaemia in a premature infant: diagnosis and treatment. *J Inherit Metab Dis* **1988**, *11*, 313-314, doi:10.1007/bf01800382.
27. Heringer, J.; Valayannopoulos, V.; Lund, A.M.; Wijburg, F.A.; Freisinger, P.; et al. Impact of age at onset and newborn screening on outcome in organic acidurias. *J Inherit Metab Dis* **2016**, *39*, 341-353, doi:10.1007/s10545-015-9907-8.
28. Hu, Z.; Yang, J.; Hu, L.; Zhao, Y.; Zhang, C.; et al. [Screening and clinical analysis of isovaleric acidemia newborn in Zhejiang province]. *Zhejiang Da Xue Xue Bao Yi Xue Ban* **2020**, *49*, 556-564, doi:10.3785/j.issn.1008-9292.2020.10.02.
29. Ito, T.; Kidouchi, K.; Sugiyama, N.; Kajita, M.; Chiba, T.; et al. Liquid chromatographic-atmospheric pressure chemical ionization mass spectrometric analysis of glycine conjugates and urinary isovalerylglycine in isovaleric acidemia. *J Chromatogr B Biomed Appl* **1995**, *670*, 317-322, doi:10.1016/0378-4347(95)00174-3.
30. James, P.M.; Levy, H.L. The clinical aspects of newborn screening: importance of newborn screening follow-up. *Ment Retard Dev Disabil Res Rev* **2006**, *12*, 246-254, doi:10.1002/mrdd.20120.
31. Kahler, S.G.; Sherwood, W.G.; Woolf, D.; Lawless, S.T.; Zaritsky, A.; et al. Pancreatitis in patients with organic acidemias. *J Pediatr* **1994**, *124*, 239-243, doi:10.1016/s0022-3476(94)70311-6.
32. Kasapkara, C.S.; Ezgu, F.S.; Okur, I.; Tumer, L.; Biberoglu, G.; et al. N-carbamylglutamate treatment for acute neonatal hyperammonemia in isovaleric acidemia. *Eur J Pediatr* **2011**, *170*, 799-801, doi:10.1007/s00431-010-1362-9.
33. Kimmoun, A.; Abboud, G.; Strazeck, J.; Merten, M.; Guéant, J.L.; et al. Acute decompensation of isovaleric acidemia induced by Graves' disease. *Intensive Care Med* **2008**, *34*, 2315-2316, doi:10.1007/s00134-008-1192-7.
34. Krieger, I.; Tanaka, K. Therapeutic effects of glycine in isovaleric acidemia. *Pediatr Res* **1976**, *10*, 25-29, doi:10.1203/00006450-197601000-00005.

35. Lee, P.J.; Harrison, E.L.; Jones, M.G.; Chalmers, R.A.; Leonard, J.V.; et al. Improvement in exercise tolerance in isovaleric acidaemia with L-carnitine therapy. *J Inherit Metab Dis* **1998**, *21*, 136-140, doi:10.1023/a:1005391508917.
36. Lee, H.H.; Lee, R.S.; Lai, C.K.; Yuen, Y.P.; Siu, T.S.; et al. A novel duplication at the putative DNA polymerase alpha arrest site and a founder mutation in Chinese in the IVD gene underlie isovaleric acidaemia. *Hong Kong Med J* **2010**, *16*, 219-222.
37. Li, H.; Shao, F.; Zhou, W. Newborn screening for isovaleric acidemia: A case report of a Chinese patient with novel variants. *Mol Genet Metab Rep* **2024**, *39*, 101088, doi:10.1016/j.ymgmr.2024.101088.
38. Lin, W.D.; Wang, C.H.; Lee, C.C.; Lai, C.C.; Tsai, Y.; et al. Genetic mutation profile of isovaleric acidemia patients in Taiwan. *Mol Genet Metab* **2007**, *90*, 134-139, doi:10.1016/j.ymgme.2006.08.011.
39. Loots, D.T.; Mienie, L.J.; Erasmus, E. Amino-acid depletion induced by abnormal amino-acid conjugation and protein restriction in isovaleric acidemia. *Eur J Clin Nutr* **2007**, *61*, 1323-1327, doi:10.1038/sj.ejcn.1602648.
40. Lund, A.B.; Lund, A.M. [Isovaleric acidaemia--a rare and serious defect in the metabolism of leucine]. *Ugeskr Laeger* **2011**, *173*, 1121-1123.
41. Malbora, B.; Avcı, Z.; Hasanoğlu, A.; Alehan, F.; Özbek, N. Late onset of isovaleric acidemia presenting with bicytopenia. *Turk J Haematol* **2010**, *27*, 216-218, doi:10.5152/tjh.2010.34.
42. Martínez Hernández, I. Actualización en el diagnóstico y tratamiento de la acidemia isovalérica. In *Revista Biomédica*; Spain, Europe, 2006; Volume 17, pp. 213-223.
43. Mayatepek, E.; Kurczynski, T.W.; Hoppel, C.L. Long-term l-carnitine treatment in isovaleric acidemia. *Pediatr Neurol* **1991**, *7*, 137-140, doi:https://doi.org/10.1016/0887-8994(91)90011-9.
44. Minkler, P.E.; Stoll, M.S.; Ingalls, S.T.; Yang, S.; Kerner, J.; et al. Quantification of carnitine and acylcarnitines in biological matrices by HPLC electrospray ionization-mass spectrometry. *Clin Chem* **2008**, *54*, 1451-1462, doi:10.1373/clinchem.2007.099226.
45. Miyamoto, S.; Ntege, E.H.; Chinen, Y.; Goto, T.; Shirakawa, J.; et al. An unusual case of oral surgical management in a patient with isovaleric acidemia and schizophrenia: A case report. *Biomed Rep* **2022**, *17*, 64, doi:10.3892/br.2022.1547.
46. Ogier de Baulny, H. Management and emergency treatments of neonates with a suspicion of inborn errors of metabolism. *Semin Neonatol* **2002**, *7*, 17-26, doi:10.1053/siny.2001.0084.
47. Pascarella, A.; Rosa, M.; della Casa, R.; Andria, G.; Parenti, G. Isovaleric acidemia. *J Pediatr Endocrinol Metab* **2011**, *24*, 399.
48. Pesce, F.; Cerone, R.; Caruso, U.; Romano, C. Acute neonatal isovaleric acidaemia presented without acidosis or ketonuria. *J Inherit Metab Dis* **1991**, *14*, 111, doi:10.1007/bf01804400.
49. Pinto, A.; Daly, A.; Evans, S.; Almeida, M.F.; Assoun, M.; et al. Dietary practices in isovaleric acidemia: A European survey. *Mol Genet Metab Rep* **2017**, *12*, 16-22, doi:10.1016/j.ymgmr.2017.02.001.
50. Poorthuis, B.J.; Jille-Vlcková, T.; Onkenhout, W. Determination of acylcarnitines in urine of patients with inborn errors of metabolism using high-performance liquid chromatography after derivatization with 4'-bromophenacylbromide. *Clin Chim Acta* **1993**, *216*, 53-61, doi:10.1016/0009-8981(93)90138-t.
51. Rabier, D.; Parvy, P.; Bardet, J.; Saudubray, J.M.; Kamoun, P. Alloisoleucine in isovaleric acidaemia. *J Inherit Metab Dis* **1992**, *15*, 154-155, doi:10.1007/bf01800358.
52. Rakheja, D.; Bober, M.B.; Fisher, S.L.; Jones, P.M. A neonate with hyperammonemia. *Lab Medicine* **2005**, *36*, 292-295, doi:10.1309/1MHJUVCBUDXVVRGC.
53. Ravikumar, A.; Abdelgani, A.; Pawlak, T.; Raphael, R.; Abdalla, A. Acute Metabolic Decompensation of Isovaleric Acidemia Presenting as Persistent Metabolic Acidosis in a Middle-Aged Man: A Case Report. *Cureus* **2024**, *16*, e67253, doi:10.7759/cureus.67253.
54. Roe, C.R.; Millington, D.S.; Maltby, D.A.; Kahler, S.G.; Bohan, T.P. L-carnitine therapy in isovaleric acidemia. *J Clin Invest* **1984**, *74*, 2290-2295, doi:10.1172/jci111657.

55. Sahai, I.; Zytkowicz, T.; Rao Kotthuri, S.; Lakshmi Kotthuri, A.; Eaton, R.B.; et al. Neonatal Screening for Inborn Errors of Metabolism Using Tandem Mass Spectrometry: Experience of the Pilot Study in Andhra Pradesh, India. *Indian J Pediatr* **2011**, *78*, 953-960.
56. Salamino, F.; Di Lisa, F.; Burlina, A.B.; Menabŏ, R.; Barbato, R.; et al. Involvement of erythrocyte calpain in glycine- and carnitine-treated isovaleric acidemia. *Pediatr Res* **1994**, *36*, 182-186, doi:10.1203/00006450-199408000-00008.
57. Sogut, A.; Acun, C.; Aydin, K.; Tomac, N.; Demirel, F.; et al. Isovaleric acidaemia: cranial CT and MRI findings. *Pediatr Radiol* **2004**, *34*, 160-162, doi:10.1007/s00247-003-1049-8.
58. Szymańska, E.; Jezela-Stanek, A.; Bogdańska, A.; Rokicki, D.; Ehmke Vel Emczyńska-Seliga, E.; et al. Long Term Follow-Up of Polish Patients with Isovaleric Aciduria. Clinical and Molecular Delineation of Isovaleric Aciduria. *Diagnostics (Basel)* **2020**, *10*, doi:10.3390/diagnostics10100738.
59. Tajima, G.; Sakura, N.; Yofune, H.; Dwi Bahagia Febriani, A.; Nishimura, Y.; et al. Establishment of a practical enzymatic assay method for determination of isovaleryl-CoA dehydrogenase activity using high-performance liquid chromatography. *Clin Chim Acta* **2005**, *353*, 193-199, doi:10.1016/j.cccn.2004.11.007.
60. Tan, J.Q.; Chen, D.Y.; Mo, Z.Q.; Li, Z.T.; Huang, J.W.; et al. [Pancytopenia and metabolic decompensation in a neonate]. *Zhongguo Dang Dai Er Ke Za Zhi* **2016**, *18*, 1150-1153, doi:10.7499/j.issn.1008-8830.2016.11.019.
61. Tokatli, A.; Coşkun, T.; Ozalp, I. Isovaleric acidemia. Clinical presentation of 6 cases. *Turk J Pediatr* **1998**, *40*, 111-119.
62. Truscott, R.J.; Malegan, D.; McCairns, E.; Burke, D.; Hick, L.; et al. New metabolites in isovaleric acidemia. *Clin Chim Acta* **1981**, *110*, 187-203, doi:10.1016/0009-8981(81)90348-x.
63. Tsai, A.C.; Lin, H.T.; Chou, M.; Bolen, J.; Zimmerman, C.; et al. Compound heterozygote variants: c.848A > G; p.Glu283Gly and c.890C > T; p.Ala297Val, of Isovaleric acid-CoA dehydrogenase (IVD) gene causing severe Isovaleric acidemia with hyperammonemia. *Mol Genet Metab Rep* **2022**, *31*, 100859, doi:10.1016/j.ymgmr.2022.100859.
64. Van Hove, J.L.; Kahler, S.G.; Millington, D.S.; Roe, D.S.; Chace, D.H.; et al. Intravenous L-carnitine and acetyl-L-carnitine in medium-chain acyl-coenzyme A dehydrogenase deficiency and isovaleric acidemia. *Pediatr Res* **1994**, *35*, 96-101, doi:10.1203/00006450-199401000-00020.
65. Vockley, J.; Ensenauer, R. Isovaleric acidemia: new aspects of genetic and phenotypic heterogeneity. *Am J Med Genet C Semin Med Genet* **2006**, *142c*, 95-103, doi:10.1002/ajmg.c.30089.
66. Wasant, P.; Liammongkolkul, S.; Kuptanon, C.; Vatanavicharn, N.; Sathienkijakanchai, A.; et al. Organic acid disorders detected by urine organic acid analysis: twelve cases in Thailand over three-year experience. *Clin Chim Acta* **2008**, *392*, 63-68, doi:10.1016/j.cca.2008.02.015.
67. Wei, C.C.; Lin, W.D.; Tsai, F.J.; Wu, J.Y.; Peng, C.T.; et al. Isovaleric acidemia diagnosed promptly by tandem mass spectrometry: report of one case. *Acta Paediatr Taiwan* **2004**, *45*, 236-238.
68. Weinberg, G.L.; Laurito, C.E.; Geldner, P.; Pygon, B.H.; Burton, B.K. Malignant ventricular dysrhythmias in a patient with isovaleric acidemia receiving general and local anesthesia for suction lipectomy. *J Clin Anesth* **1997**, *9*, 668-670, doi:10.1016/s0952-8180(97)00187-6.
69. Wilson, W.G.; Audenaert, S.M.; Squillaro, E.J. Hyperammonaemia in a preterm infant with isovaleric acidaemia. *J Inherit Metab Dis* **1984**, *7*, 71, doi:10.1007/bf01805807.
70. Yudkoff, M.; Cohn, R.M.; Puschak, R.; Rothman, R.; Segal, S. Glycine therapy in isovaleric acidemia. *J Pediatr* **1978**, *92*, 813-817, doi:10.1016/s0022-3476(78)80164-4.
71. Zaki, O.K.; Priya Doss, C.G.; Ali, S.A.; Murad, G.G.; Elashi, S.A.; et al. Genotype-phenotype correlation in patients with isovaleric acidaemia: comparative structural

modelling and computational analysis of novel variants. *Hum Mol Genet* **2017**, *26*, 3105-3115, doi:10.1093/hmg/ddx195.
72. Zegarra Buitron, E.; Vidal Panduro, D.A.; Guillén Ramírez, N.S.; González Arteaga, M. Isovaleric Acidemia: A Case Report. *Cureus* **2023**, *15*, e49362, doi:10.7759/cureus.49362.
